# Immune Checkpoint Inhibitors in the Treatment of Advanced Melanoma in Older Patients: An Overview of Published Data

**DOI:** 10.3390/cancers17111835

**Published:** 2025-05-30

**Authors:** Marko Lens, Jacob Schachter

**Affiliations:** Ella Lemelbaum Institute for Immuno Oncology, Chaim Sheba Medical Center, Tel Aviv 6997801, Israel

**Keywords:** melanoma, geriatric, older, immunotherapy, immune checkpoint inhibitors, systemic therapy

## Abstract

Melanoma has important burden in older populations due to high incidence and aggressive biology. The emergence of immunotherapy with immune checkpoint inhibitors and targeted therapy (BRAF/MEK inhibitors) significantly improved melanoma prognosis. Currently, the body of knowledge on the efficacy and tolerability of these treatments in geriatric patients is primarily based on the results outside of clinical trials since the majority of clinical studies do not include older patients. We present a comprehensive narrative review of published data regarding efficacy and safety of therapeutic modalities using immune checkpoint inhibitors in patients age 65–75 years and >75 years: the anti-cytotoxic T lymphocyte-associated protein 4 (CTLA-4) inhibitor (ipilimumab), the anti-programmed death-ligand 1 (PD-1) inhibitors (nivolumab and pembrolizumab), and the lymphocyte activation gene-3 (LAG-3) inhibitor (relatlimab). We carefully address difficulties in multi-disciplinary clinical decision-making in care of older melanoma patients. Although many older patients may not be offered immunotherapy, the available evidence indicates that immunotherapy is equally beneficial in the older patients and does not have higher incidence of adverse events in this group of patients compared to younger population.

## Simple Summary

Treatment of stage III/IV melanoma in geriatric patients is challenging in daily clinical practice. Many older melanoma patients are not offered the most commonly prescribed systemic therapy regimens that are regularly offered to younger patients. In the last decades, the use of immune checkpoint inhibitors (ICIs) dramatically improved survival of melanoma patients. However, due to the fact that older patients were considerably under-represented in randomised clinical trials evaluating efficacy and safety if ICIs, data on the use of ICIs in this age subgroup are still limited. The objective of our review was to conduct an overview of published evidence on the clinical management of older melanoma patients treated with ICIs (monotherapy or combined regimens). We evaluated clinical trials that assessed three different types of ICIs: CTLA-4 inhibitor (ipilimumab), PD-1 inhibitor (pembrolizumab and nivolumab), and lymphocyte activation gene- 3 (LAG-3) inhibitor (relatlimab). Currently available evidence suggests no difference in the efficacy of ICIs and frequency of treatment-related adverse events in older melanoma patients compared to patients from younger age groups. Thus, stage III/IV melanoma patients from the geriatric population should be offered the same systemic therapy with ICIs as melanoma patients aged < 65 years.

## 1. Introduction

The growing cancer burden among people aged 80 years or over represents a major challenge for healthcare systems worldwide [1]. In older generations, cutaneous melanoma poses a significant worldwide public health concern. According to recent statistics, the incidence of melanoma has either declined or stabilised among young and middle-aged individuals, while it is still increasing among the old population [2,3]. As people age, their risk in developing melanoma rises. Recently published data showed that in the United States during the period 2017–2019, the probability of developing melanoma in age group 64 to 85 years was 1 in 23 in men and 1 in 92 in women, while in the age group ≥ 85 years it was 1 in 73 and 1 in 188, respectively [4]. Statistics from the Surveillance, Epidemiology, and End Results (SEER) program for the period 2017–2022 show that 26.9% of melanoma was detected in the age group 65–74 years, while 27.5% of melanoma cases were diagnosed in people 75 years or older [5]. Although effective prevention strategies and new treatment regimens led to notable improvements in mortality and survival in younger populations, older age groups have seen an increase in mortality [6,7,8]. From 1999 to 2021, the United States Cancer Statistics (USCS) database revealed that the five-year relative survival rates for invasive cutaneous melanomas were 95.7% (95% CI: 95.5–95.9) for people under 45 years and 91.0% (95% CI: 90.4–91.6) for those ≥75 years [9].

In the past, patients with advanced melanoma had poor prognosis with a median survival of about 6 months [10]. However, in the last decade the use of targeted therapy (combination of BRAF/MEK inhibitors) for BRAF-mutant melanoma and immunotherapy–immune checkpoint inhibitors blocking programmed cell death protein 1 (PD-1) and/or cytotoxic T-lymphocyte-associated protein 4 (CTLA-4) has transformed the treatment landscape and dramatically improved the survival rate of patients with advanced melanoma [11,12]. Recently, the third clinically relevant immune checkpoint inhibitor blocking the lymphocyte activation gene-3 (LAG-3) was approved for melanoma treatment [13]. Current immunotherapeutic options include anti-PD-1 monotherapy with nivolumab, pembrolizumab and combination immunotherapy with nivolumab plus anti-CTLA-4 agent ipilimumab, or nivolumab and anti-LAG-3 agent relatlimab [14]. For patients with advanced melanoma, combined immunotherapy is now regarded as a first-line treatment [15,16].

Despite the fact that most melanoma cases are diagnosed in people 65 years of age or older, data regarding the safety and effectiveness of immunotherapy for this subgroup of patients are limited because older patients are highly under-represented in clinical trials assessing targeted therapy and immunotherapy [17]. Thus, it is challenging to establish the actual impact of immunotherapy in older melanoma patients. It has been suggested that immunotherapy may be less efficient in older patients due to the immunosenescence, a physiological process of age-related decline of immunity [18,19]. However, research data suggest that the anti-tumour response in older melanoma patients is not negatively impacted by age-related decline in immune function [20,21,22].

In routine clinical practice, each older patient with advanced melanoma should undergo a comprehensive geriatric assessment. This multidimensional assessment includes evaluation of the overall health status composed of various clinically relevant domains: functional status, comorbidity, cognition, psychological status, nutritional conditions, medications, economic status and social support [23,24]. Geriatric assessment is an integral part of the treatment decision-making process, which also includes an advance care planning of the treatment regimen and follow-up, prediction of malignancy-related outcomes and the risk of treatment-related toxicities, and development of supportive care during oncology treatments [25,26,27].

**Figure 1:**
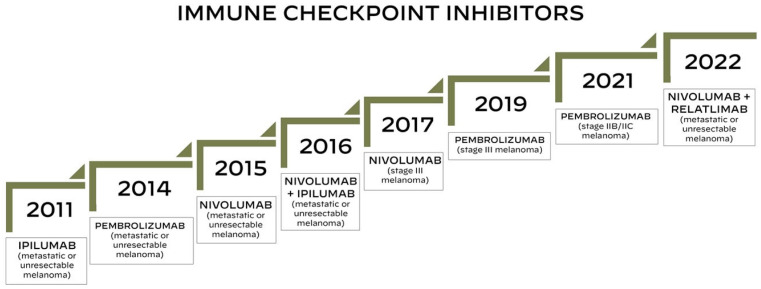
Timeline of the US FDA approvals of immune checkpoint inhibitors for the treatment of melanoma.

Currently three different types of immune checkpoint inhibitors (ICIs) are approved for the treatment of advanced melanoma: CTLA-4 inhibitor (ipilimumab), PD-1 inhibitor (pembrolizumab and nivolumab) and LAG-3 inhibitor (relatlimab) [13,28,29]. Figure 1 represents a timeline of the US FDA approvals of immune checkpoint inhibitors used for the treatment of melanoma.

The aim of the present narrative review is to provide a comprehensive summary of the published clinical evidence and available knowledge on the management of advanced melanoma in the older population, with a particular focus on the use of immunotherapeutic approaches with immune checkpoint inhibitors.

## 2. CTLA-4 Inhibitor (Ipilimumab)

Ipilimumab is a fully human, monoclonal antibody that blocks the cytotoxic T lymphocyte-associated antigen 4 (CTLA-4) signalling, which induces an unrestrained T-cell activation and proliferation and amplifies T-cell-mediated immunity (anti-tumour immune response) [30,31]. It was the first checkpoint inhibitor approved by the US FDA in 2011 for the treatment of unresectable metastatic melanoma at the dose of 3 mg/kg.

Ipilimumab was approved based on the results from the phase 3 randomised trial (MDX010-20) including a total of 676 patients with unresectable stage III or IV melanoma who were randomly assigned in a 3:1:1 ratio to receive ipilimumab plus gp100, ipilimumab alone, or gp100 alone [32]. In this trial, 29% of enrolled patients (196 patients) had age ≥ 65 years, of which 78.6% received ipilimumab (57.2% ipilimumab plus gp100 and 21.4% ipilimumab alone). Patients ≥ 65 years treated with ipilimumab monotherapy (3 mg per kilogram) had significantly better overall survival (OS) than those who received gp100 (HR = 0.61, 95% CI 0.38–0.99). Subsequent analysis of the time to onset and resolution of immune-related adverse events (irAEs) associated with ipilimumab therapy did not reveal a difference in the incidence of these events between patients ≥ 65 years and those < 65 years [33].

In a randomised, double-blind, phase 3 study (CA184-024 trial), the efficacy of ipilimumab was evaluated by comparing therapy with ipilimumab (10 mg per kilogram) in combination with dacarbazine versus treatment with dacarbazine plus placebo in 502 patients with untreated stage III (unresectable) or stage IV melanoma with measurable lesions [34]. Of all patients included, 31.9% were ≥ 65 years. While the trial’s results indicated that patients treated with ipilimumab plus dacarbazine had a significantly longer survival than those treated with dacarbazine plus placebo (HR = −0.33, 95% CI −0.53 to −0.14), the survival benefit did not reach statistical significance in a subgroup analysis of patients aged ≥ 65 years (HR = −0.09, 95% CI −0.44 to −0.25).

Results from the EORTC 18071 (CA184-029) trial showed that high-risk patients with stage III cutaneous melanoma (excluding lymph node metastasis ≤ 1 mm or in-transit metastasis) who received ipilimumab (10 mg/kg) in an adjuvant setting after complete resection of lymph nodes had significantly longer recurrence-free survival (RFS), distant metastasis-free survival (DMFS), and OS at a median follow-up of 5.3 years [35,36]. However, a subgroup analysis that included patients ≥ 65 years (17.7% of all enrolled patients) failed to show that patients who received adjuvant ipilimumab had benefit in RFS and OS in comparison to placebo-treated patients (HR = 0.80, 95% CI 0.49–1.30 and HR = 0.88, 95% CI 0.50–1.56, respectively) [36]. No difference in the incidence of immune-related adverse events was observed between patients ≥ 65 years and those with age < 65 years, despite the fact that 52% of all patients who received ipilimumab discontinued treatment during the induction phase (the first four doses) [37].

At the median follow-up of 61 months, a phase 3 randomised controlled trial (CA184-169) comprising 727 patients with previously treated or untreated unresectable stage III or IV melanoma demonstrated that the ipilimumab monotherapy at 10 mg/kg resulted in significantly longer OS than the treatment with ipilimumab administered at 3 mg/kg [38,39]. Patients ≥ 65 years who received ipilimumab at 10 mg/kg (38.6% of all patients receiving high-dose ipilimumab) had a median survival of 10.8 months, whereas those who received ipilimumab at 3 mg/kg (42.5 percent of all patients receiving low-dose ipilimumab) had a median survival of 11.5 months. However, no significant difference in the long-term survival benefit between patients receiving high-dose and low-dose ipilimumab monotherapy was found in a subgroup analysis of patients ≥ 65 years (HR = 0.97, 95% CI 0.75–1.25). Although this trial showed that ipilimumab treatment at 10 mg/kg was associated with more frequent irAEs than treatment at 3 mg/kg (26% versus 12%, respectively), the incidence of irAEs has not been published separately for age subgroups (patients ≥ 65 years against patients < 65 years).

In the North American Intergroup E1609 phase 3 trial including 1670 patients with resected high-risk cutaneous melanoma and unknown primary melanoma, patients who received adjuvant therapy with ipilimumab at 3 mg/kg had significantly superior OS compared to patients treated with high-dose interferon alfa (HDI) and no significant difference in RFS and OS compared to patients who received ipilimumab at 10 mg/kg [40]. This trial enrolled only 14.5% of patients > 65 years and no analysis between this age group and younger patients was provided. However, a subgroup analysis that contrasted patients > 55 years with those ≤ 55 years, while failing to show significant RFS and OS benefit of ipilimumab at 3 mg/kg in older patients (HR = 0.80, 95% CI 0.63–1.02 and HR = 0.76, 95% CI 0.56–1.04, respectively), demonstrated that this group of patients may experience a significant improvement in RFS when treated with ipilimumab at 10 mg/kg (HR = 0.78, 95% CI 0.61–0.99) [41]. Although analyses of all study participants and on 549 patients who were enrolled after the trial was modified revealed that irAEs were less common with ipilimumab at 3 mg/kg than with ipilimumab at 10 mg/kg, and that patients treated with high-dose versus low-dose ipilimumab required corticosteroid use more frequently (75.7% and 52.5%, respectively), no analysis of the frequency of irAEs and corticosteroid use by age subgroups has been published [42,43].

## 3. PD-1 Inhibitors

Pembrolizumab and nivolumab are humanised monoclonal antibodies that work by binding to the programmed death-1 (PD-1) receptor on the surface of T-cells, which inhibits interaction with its immune-suppressing ligands PD-L1 and PD-L2, thus restoring T-cell activation and anti-tumour immune responses [44].

### 3.1. Pembrolizumab

Based on the results of the randomised cohort B2 carried out within the phase 1 KEYNOTE-001 trial, Pembrolizumab was the first PD-1 inhibitor to be approved by the US FDA (September 2014), with a recommended dose of 2 mg/kg every three weeks for the treatment of patients with unresectable melanoma or metastatic melanoma who have progressed following treatment with ipilimumab or a BRAF inhibitor [45].

A non-randomised cohort B1 in the phase 1 KEYNOTE-001 trial enrolling 135 patients demonstrated the safety and anti-tumour activity of three different treatment regimens (10 mg/kg every 2 weeks, 10 mg/kg every 3 weeks, and 2 mg/kg every 2 weeks) of pembrolizumab, whereas a randomised cohort B2 comprising 173 patients with ipilimumab-refractory advanced melanoma revealed no difference in overall response rate (ORR) between patients treated with pembrolizumab at 2 mg/kg versus those who received 10 mg/kg every 3 weeks (26% in both groups) [46,47]. The incidence of drug-related adverse events was 82% in both groups, and there was no difference in OS between the two treatment groups (HR = 1.09, 95% CI 0.68–1.75). However, patients treated with pembrolizumab at 3 mg/kg had a significantly higher occurrence of toxicity grade 3 or 4 than patients treated with pembrolizumab at 10 mg/kg (15% versus 8%, respectively). Among 157 patients in cohort B2 that were fully analysed, 58 patients (36.7%) were 65 years of age or older. No difference in OSS was observed between this group versus patients < 65 years (ORR = 22.4%, 95% CI 12.5–35.3 and ORR = 28.3%, 95% CI 19.7–38.2, respectively).

In 540 patients with unresectable stage III or stage IV melanoma who had not responded to ipilimumab plus BRAF/MEK inhibitor therapy (if BRAF V600 mutant), two doses of pembrolizumab (2 mg/kg and 10 mg/kg) were compared with investigator-choice chemotherapy in the randomised phase 2 KEYNOTE-002 study [48]. A pre-specified subgroup analysis of 359 patients (35% of whom were 65 years of age or older) who received pembrolizumab 2 mg/kg versus chemotherapy and 360 patients (34.2% ≥ 65 years) who received pembrolizumab 10 mg/kg versus chemotherapy revealed no difference in achieved PFS between patients ≥65 years and those under 65 years of age (*p* = 0.71 and 0.55, respectively), despite the fact that analysis of all included patients showed that both pembrolizumab regimens significantly improved progression-free survival (PFS) versus chemotherapy. When compared to chemotherapy, pembrolizumab (2 mg/kg or 10 mg/kg) improved PFS, objective-response rate (ORR), and durability of response, but it did not significantly affect OS, according to the final analysis performed at a median follow-up of 28 months [49].

Patients who received pembrolizumab (10 mg/kg every 2 weeks or 10 mg/kg every 3 weeks) had significantly better PFS and OS than those treated with ipilimumab (3 mg/kg every 3 weeks) according to data from the randomised phase 3 KEYNOTE-006 trial, which enrolled 834 patients with unresectable stage III or IV advanced melanoma [50]. Five-year and seven-year follow-up revealed that patients treated with two pembrolizumab regimens had significant benefit in median OS compared to those who received ipilimumab (32.7 versus 15.9 months and 32.7 versus 15.9 months, respectively) [51,52]. At a 10-year follow-up, the reported 7-year median OS remained unchanged [53]. The pembrolizumab benefit was observed across two age subgroups (<65 years and ≥65 years).

Based on the results from the KEYNOTE-006 trial, in December 2015 the US FDA approved pembrolizumab at the dose 2 mg/kg every 3 weeks as the first-line treatment of patients with unresectable or metastatic melanoma, while based on the KEYNOTE-002 trial, pembrolizumab was approved for the treatment of patients with ipilimumab-refractory advanced melanoma.

According to a pooled analysis of data from three trials (KEYNOTE-001, KEYNOTE-002, and KEYNOTE-006) that included 1558 patients with advanced melanoma (42.2% of patients were 65 years or older), the majority of patients ≥ 65 years had BRAF wild-type melanoma rather than BRAF V600 mutant melanoma (83.9% versus 16.1%) [54]. In a subpopulation of patients ≥ 65 years, the overall response rate, 4-year PFS rate, and 4-year OS rate were significantly lower in patients who were previously treated with BRAF and/or MEK inhibitor compared to those who had no prior BRAF/MEK inhibitor therapy (36.2% versus 56.3%, 15.7% versus 37.3%, and 27.6% versus 56.8%, respectively). In the landmark analysis of 1567 patients in the KEYNOTE-001, KEYNOTE-002, and KEYNOTE-006 trials, there was no difference in the safety profile of pembrolizumab monotherapy among patients ≥ 65 years and those < 65 years, since both age groups experienced a similar rate of any-grade treatment-related adverse events (TRAEs) (79.9% versus 81.3%, respectively) [55].

Based on the results from the EORTC1325/KEYNOTE-054 trial, in 2019 pembrolizumab gained the US FDA approval for the adjuvant treatment of patients with melanoma with involvement of lymph nodes following complete resection (stage III melanoma). In this study, 1011 patients with completely resected, stage IIIA (>1 mm lymph node metastasis), IIIB, or IIIC melanoma were assigned to adjuvant pembrolizumab therapy or a placebo [56]. A 7-year analysis showed that patients treated with adjuvant pembrolizumab had substantially improved RFS, distant-metastasis free survival (DMFS), and progression/recurrence-free survival 2 (PRFS2) compared to those who received the placebo (50% vs. 36%, 54% vs. 42%, and 61% vs. 53%, respectively) [57]. However, this trial failed to detect any difference in the treatment benefit among different age groups (<65 years versus ≥65 years). This trial confirmed an association between irAEs and treatment outcome: the efficacy of adjuvant pembrolizumab was higher after the occurrence of irAEs than before or no onset of irAEs [58].

In 2021, the US FDA approved pembrolizumab for the adjuvant treatment of patients with stage IIB or IIC melanoma following complete resection, based on the findings of the KEYNOTE-716 trial, which enrolled 976 patients (38.7% ≥ 65 years) with completely resected, high-risk, stage IIB or IIC melanoma. This study confirmed that, in comparison to the placebo adjuvant, pembrolizumab significantly decreased the risk of recurrence (*p* = 0.0066) and improved DMFS (*p* = 0.0029) [59,60]. No efficacy difference was detected between the two age groups (patients ≥ 65 years and those <65 years).

### 3.2. Nivolumab

In 2015, nivolumab was the second PD-1 inhibitor to receive US FDA approval for the treatment of patients with unresectable or metastatic melanoma based on the results of the randomised phase 3 CheckMate 037 trial, which randomly assigned 405 patients in a 2:1 ratio to receive nivolumab 3 mg/kg every 2 weeks or ICC (dacarbazine 1000 mg/m^2^ every 3 weeks or paclitaxel 175 mg/m^2^ combined with carboplatin area under the curve 6 every 3 weeks) [61]. Patients treated with nivolumab had a significantly higher objective response rate than those treated with ICC (31.7% versus 10.6%, respectively), according to the first analysis of the data from patients with unresectable stage IIIC or IV metastatic melanoma, who had either progressed after anti-CTLA-4 plus BRAF inhibitor treatment (if BRAF V600 mutant melanoma) or who had failed prior anti-CTLA-4 treatment (if BRAF WT melanoma) [62]. When comparing patients treated with nivolumab to those treated with ICC, the CheckMate 037 study did not demonstrate an OS or PFS advantage [63]. Although this trial did not report notable differences in OS in a pre-specified subgroup analysis, an HR of > 1.10 was observed for patients younger than 65 years.

A randomised phase 3 CheckMate 066 trial including 418 previously untreated patients with stage III or IV melanoma without a BRAF mutation found that patients treated with nivolumab had a significantly higher objective response rate and 1-year survival rate than patients who received dacarbazine, 40% versus 13.9% and 72.9% versus 42.1%, respectively [64]. In three age-subgroups (47.9% < 65 years, 36.1% 65–75 years, and 16% ≥ 75 years), an unstratified analysis of OS showed that the hazard of mortality decreased with age (HR = 0.52, HR = 0.44, and HR = 0.25, respectively). The significant OS benefit of nivolumab over dacarbazine (39% versus 17%, respectively) was validated by a 5-year analysis [65].

In 2017, the US FDA approved nivolumab as the first PD-1 checkpoint inhibitor for the adjuvant treatment of patients with melanoma with lymph node involvement or in patients with metastatic disease who had undergone complete resection. This approval was based on a randomised, phase 3 CheckMate 238 trial that evaluated nivolumab (3 mg/kg every 2 weeks) versus ipilimumab (10 mg/kg every 3 weeks) in 906 patients with resected stage IIIB, IIIC, or IV melanoma. Adjuvant nivolumab provided significantly better RFS and lower incidence of grade 3–4 adverse events than ipilimumab (70.5% versus 60.8% and 45.9% versus 14.4%, respectively) at 12 months [66]. While there was no difference in OS between the two treatment groups at the 4-year follow-up, nivolumab significantly improved RFS when compared to ipilimumab (51.7% versus 41.2%) [67]. In the CheckMate 238 trial, the efficacy of nivolumab was statistically significant for patients < 65 years (HR = 0.72, 95% CI 0.58–0.89), while in the older group of patients (25.8% of all included patients with age ≥ 65 years), the efficacy was not clearly significant (HR = 0.72, 95% CI 0.51–1.00).

The US FDA approved nivolumab 240 mg every 2 weeks or 480 mg every 4 weeks for the adjuvant treatment of completely resected stage IIB/C melanoma in patients 12 years and older due to the statistically significant improvement in RFS seen in the nivolumab arm of the phase 3 CheckMate 76K trial (89% versus 79% in placebo), which enrolled 790 patients [68]. In this trial, which included 41.8% of patients ≥ 65 years, no difference in efficacy and safety was detected between the various age groups.

### 3.3. Combination Therapy: CTLA-4 Inhibitor and PD-1 Inhibitor

Although single-agent immune checkpoint inhibitor (ICI) therapy with monoclonal antibodies targeting CTLA-4 and PD-1 has been the standard of care for patients with metastatic melanoma, the majority of patients did not experience long-term benefits from ICI monotherapy. Researchers examined several combination regimens to overcome resistance to immune checkpoint blockade, enhance anti-tumour immune response, and improve treatment efficacy while minimising toxicity [69,70].

The first combination of immune checkpoint inhibitors (nivolumab in combination with ipilumab) for the treatment of unresectable or metastatic melanoma was approved by the US FDA in 2016 based on the results from the randomized, phase 3 trial CheckMate 067. This study, enrolling 945 patients with unresectable stage III or IV melanoma, evaluated the efficacy and safety of three treatment regimens: (1) nivolumab (1 mg/kg) plus ipilimumab (3 mg/kg) every 3 weeks for 4 doses followed by nivolumab (3 mg/kg) every 2 weeks; or (2) nivolumab alone (3 mg/kg) every 2 weeks; or (3) ipilimumab alone (3 mg/kg) every 3 weeks for 4 doses [71]. In the trial, there were 565 patients (59.8%) aged < 65 years, 262 patients in the age group ≥ 65– < 75 years, and 118 patients (12.5%) aged ≥ 75 years. Patients treated with nivolumab plus ipilimumab had a significantly greater median PFS (11 months) than those treated with either nivolumab or ipilimumab alone (6.9 months and 2.9 months, respectively). However, patients treated with a combination of immune checkpoint inhibitors experienced more frequent grade 3–4 adverse events (55%) compared to patients receiving nivolumab alone or ipilumab alone (16.3% and 27.3%, respectively).

According to the final 10-year results of the CheckMate 067 trial, patients treated with nivolumab plus ipilimumab and those treated with nivolumab monotherapy had better OS (median OS of 71.9 months and 36.9 months, respectively) than patients treated with ipilimumab alone (median OS of 19.9 months) [72]. A subgroup analysis (patients < 65 years versus patients ≥ 65 years) detected a statistically significant difference in melanoma-specific survival in patients treated with nivolumab plus ipilimumab versus nivolumab in both age groups (HR = 0.45, 95% CI 0.34–0.60 and HR = 0.52, 95% CI 0.37–0.72, respectively). The same results were observed for nivolumab versus ipilimumab monotherapy in both age groups (HR = 0.58, 95% CI 0.44–0.75 and HR = 0.52, 95% CI 0.62–0.86, respectively). However, this subgroup analysis failed to show a statistically significant difference in melanoma-specific survival in two age groups when treated with nivolumab plus ipilimumab versus nivolumab.

A phase IIIb/IV CheckMate 511 study assessed the safety profile of nivolumab 3 mg/kg plus ipilimumab 1 mg/kg in 180 patients (NIVO3 + IPI1 group) versus nivolumab 1 mg/kg plus ipilimumab 3 mg/kg in 178 patients (NIVO1 + IPI3 group) [73]. Although a high rate of treatment discontinuation was observed in the NIVO1 + IPI3 group due to more frequent adverse events, this study did not reveal any difference in the safety profile among patients ≥ 65 years compared to those with an age < 65 years (35.1% versus 64.9%% in NIVO3 + IPI1 group and 32.6% versus 67.4% in NIVO1+IPI3 group). However, data pooled from six clinical trials (CheckMate 003, 004, 066, 067, 069, and 511), including a total of 1375 patients at the median follow-up of 43.6 months, showed that the OS was significantly better in patients receiving NIVO + IPI than in patients on NIVO monotherapy (HR = 0.78, 95% CI 0.67 to 0.91) [74]. A multivariate analysis of 301 patients ≥ 65 years versus 497 patients age <65 years found that the OS was significantly longer in patients age <65 years treated with NIVO + IPI (HR = 1.36, (6% CI 1.10 to 1.69, *p* = 0.0051). Furthermore, a classification and regression tree (CART) analysis revealed that the most important clinical factor determining worst survival for patients treated with NIVO + IPI was age ≥ 65 years combined with a lactate dehydrogenase (LDH) level above the upper limit of normal (250 U/L).

## 4. LAG-3 Inhibitor (Relatlimab)

In 2022, the US FDA approved nivolumab and the LAG-3-blocking antibody relatlimab for the treatment of unresectable or metastatic melanoma based on the results from the RELATIVITY-047 trial. This phase 3 trial enrolling 716 patients evaluated the efficacy of relatlimab and nivolumab as a fixed-dose combination versus nivolumab alone [75]. Patients treated with the relatlimab–nivolumab combination had significantly better PFS compared to those who received nivolumab monotherapy (10.1 months versus 4.6 months, *p* = 0.006). Subgroup analysis demonstrated that patients aged ≥ 65 years (46.4% of all patients) treated with the relatlimab–nivolumab combination had significantly longer PFS than those treated with nivolumab alone (HR = 0.69, 95% CI 0.51–0.93). However, when analysing a subgroup of patients aged ≥ 75 years (17.6% of all patients), although PFS favoured relatlimab–nivolumab combination over nivolumab alone, statistical significance was not detected.

An exploratory, post hoc indirect treatment comparison (ITC) assessing published data from the RELATIVITY-047 trial (339 patients treated with nivolumab plus relatlimab) and CheckMate 067 trial (297 patients treated with nivolumab plus ipilimumab) failed to show significant differences in OS, PFS, ORR, and melanoma-specific survival (MSS) [76]. However, patients treated with nivolumab plus relatlimab experienced less frequent grade 3–4 treatment-related adverse events (TRAEs) and any-grade TRAEs that led to treatment discontinuation, when compared to those who received nivolumab plus ipilimumab (23% versus 61% and 17% versus 41%, respectively). Subgroup analysis did not show any difference in the efficacy between different aged groups.

## 5. Discussion

Patients with melanoma currently have a wide range of treatment options. The introduction of monoclonal antibodies targeting cytotoxic T lymphocyte antigen-4 (CTLA-4), program death protein 1 (PD-1), or lymphocyte activation gene-3 (LAG-3) dramatically changed the prognosis of patients with late-stage melanoma.

Currently available clinical data from randomised controlled trials, however, failed to show significant difference in OS between older melanoma patients (age ≥ 65 years) and younger patients (age < 65 years) treated with immune checkpoint inhibitors. However, it is important to emphasise that in registration-randomised trials, the included older patients were very fit because of the stringent inclusion and exclusion criteria. Thus, the results of these trials may therefore not be representative for the actual older population; thus, their use should be carefully evaluated.

Our overview of currently published studies confirms that data specific for the use of immunotherapy in the treatment of advanced melanoma for patients aged > 65 years are still limited. Thus, the clinical management of older patients remains challenging. In routine daily practice, many older patients receive systemic therapy less frequently than younger patients, although clinical results from randomised clinical trials confirmed similar efficacy and safety of immune checkpoint inhibitors in the geriatric population compared to the younger counterpart.

Results of a study conducted on 2012 patients using the Surveillance, Epidemiology, and End Results (SEER) Registry, linked with the Medicare database, showed important changes in the frequency of use of systemic therapy in melanoma patients > 65 years. In the period between 2008 and 2010, about 28.6% stage III melanoma patients and 35.5% stage IV melanoma patients received systemic therapy; in the period from 2015 to 2019, the percentage of stage III and stage IV melanoma patients receiving therapy almost doubled (55.4% and 68.0%, respectively) [77]. This study also revealed a significant shift in the type of systemic therapy used for the treatment of stage III and IV melanoma patients above 65 years: from 2008 to 2010 the standard first-line systemic treatment was chemotherapy or cytokines, while by 2015 to 2019 the majority of patients received PD-1 inhibitors alone or in combination with ipilimumab.

Actual data from different national cohorts supported the use of immune checkpoint inhibitors (ICIs) in melanoma patients aged 65 years and older, showing significant improvement in survival and acceptable treatment tolerability in this group of patients [78,79].

A population-based study conducted on 1435 melanoma patients from the SEER—Medicare database, treated with ICIs (ipilimumab, PD-1 inhibitors, or combination ipilimumab plus PD-1 inhibitor) in the period from 2012 to 2015, found that older melanoma patients treated with PD-1 inhibitors had prolonged survival compared to those treated with ipilimumab [80].

Data from a prospective cohort including 3054 patients with unresectable stage IIIc or IV melanoma collected from the Dutch Melanoma Treatment Registry (DMTR) between 2013 and 2017 demonstrated that melanoma patients with age ≥ 75 years were treated less frequently than younger patients [81]. However, analysis of the same data found that these older patients when treated with systemic therapy had no difference in toxicity compared to younger patients; they had a borderline statistically significant decrease in melanoma-specific survival.

Results from another cohort study using the DMTR data from 885 patients aged ≥ 65 years with resected stage III or IV cutaneous melanoma treated with adjuvant anti-PD-1 therapy in the period from 2018 until 2022, showed that older patients had similar recurrence-free survival (RFS) to the one observed in the same age group in previously conducted clinical trials [82].

A meta-analysis of 15 phase 3 randomised trials evaluating ICIs therapy for different types of malignancies, including patients ≥ 75 years, confirmed the survival benefit of ICIs in melanoma when used as the first-line treatment [83].

However, melanoma management with ICIs is associated with toxicity, and many patients develop immune-related adverse events (irAEs).

In a retrospective cohort including 773 patients, Fletcher et al. observed that while older melanoma patients have a similar occurrence rate of adverse events as younger patients, patients above 80 years of age have lower treatment tolerability and thus have more common treatment cessation compared to younger patient subgroups [84].

A study on 4489 melanoma patients aged ≥ 65 years from the SEER—Medicare database treated with ICIs from 2011 to 2015 observed that the use of ICIs is associated with the increased risk of irAEs [85]. However, findings from this population-based cohort were consistent with the occurrence of irAEs observed in clinical trials evaluating efficacy and toxicity of ICIS.

In the first prospective study (ELDERS) assessing the safety of immunotherapy in 140 cancer patients, Gomes et al. found no evidence that the incidence irAEs of grade ≥ 3 was higher in older patients (≥70 years) receiving checkpoint inhibitors [86].

In another prospective observational study including 92 stage III and IV melanoma patients (age ≥ 70 years) who received anti–PD-1 monotherapy, grade ≥ 3 irAEs occurred in 20% of patients [87]. This study observed no difference in the frequency of grade ≥ 3 irAEs in fit and frail older patients, although this group of patients had to be hospitalised and experienced an increased length of hospitalisation due to development of irAE [87].

Actual data from a DMTR cohort revealed that patients aged ≥ 65 years with resected stage III/IV melanoma with multiple comorbidities had increased risk for the development of grade 3 or 4 irAEs [81,82].

It has been suggested that older patients are at increased risk of developing treatment-induced adverse events due to age-related changes in pharmacokinetics and pharmacodynamics, increasing comorbidity and use of different medications [88]. The SEER—Medicare data from 4519 older patients with stage III/IV showed that 85% of patients had multimorbidity [89].

A retrospective observational study enrolling 2216 patients aged ≥ 65 years with stage IV melanoma registered at DMTR between July 2013 and March 2020 found that toxicity associated with checkpoint inhibitors in the actual population was comparable to that observed in randomised clinical trials. However, data from this study failed to demonstrate that the toxicity related to treatment with checkpoint inhibitors increases with comorbidity [90].

## 6. Conclusions

Although many older patients may not be offered immunotherapy, to-date there is no evidence suggesting that older patients should be treated any differently from younger stage III/IV melanoma patients receiving ICIs. In fact, this overview of published data indicates that immunotherapy is equally beneficial in older patients, and that it does not have a higher incidence of adverse events in this group of patients when compared to the younger population. However, due to the lack of data from randomised trials, a properly designed randomised controlled trial enrolling only patients aged ≥ 65 years should be conducted to give us further insights into the efficacy, toxicity, and quality of life of different treatment protocols using ICIs.
